# Observations on Hare-Lip and Cleft Palate

**Published:** 1876-12

**Authors:** 


### OBSERVATIONS ON HARE-LIP AND CLEFT PALATE.

Sir William Fergusson, in an article on operations for the cure of these deformities, mentions the case of a young girl, six years of age, on whom an operation for double hare-lip had been performed during infancy. The prominence of the intermaxillary bones had been bent back into the cleft existing in the palate. There was still considerable deformity existing, for remedying which a second operation was performed. In doing this the intermaxillary portion was removed, and, being macerated, showed that by having been placed in its new position at the time of the first operation, the front surface had become the under one, and consequently the teeth, in developing, had assumed a horizontal instead of a vertical position. When this part projects, Sir William is in the habit of cutting and raising the mucus membrane before removing the bone, thereby leaving the membrane as a soft cushion and a more complete covering to the side not interfered with. In perfecting his recent improvement on the operation for closure of cleft in the hard palate, he has found that in many cases stitches may not be necessary. He says; "I have found again and again that, when the edges of the gap have been pared and the chisel introduced, it answers to cause approximation of the raw margins by stopping the opening made on the hard substance by the chisel, with lint, so as to make the desired closure in the centre. The pledgets of lint keep the parts as steady as, if not more so than the stitches, whilst they obviate the necessity of the additional injury of the stitch-punctures. This practice, I am confident, is available in all instances when the opening in the bones is of brief extent, as the front stitch in the soft palate may be put in close behind the posterior margin of the bones."

He describes a very useful gag for keeping the mouth open during operations on the palate or any part of the mouth. The two legs or blades of the instrument which pry apart the teeth are connected by a hinge with two powerful handles. The instrument can be kept open at any desired width by means of a screw and button. This seems a much more useful and simple instrument than any gag hitherto devised.

Mr. Francis Mason has been treating lately, at St. Thomas's Hospital, a number of patients with cleft palate by applications of strong nitric acid. The ages of the patients vary from a few weeks to several years. He thinks this method of effecting union is especially applicable to cases in which the cleft is of average extent, and even where the hard palate is partially implicated. The application is attended with no pain or inconveniences whatever to the patient. Other caustics were tried, but nitric acid was preferred. Such cases can be treated as out-patients. The results of this method of treatment have not yet been given, however. —*Boston Medical and Surgical Journal.*

